# Correlation between Dengue-Specific Neutralizing Antibodies and Serum Avidity in Primary and Secondary Dengue Virus 3 Natural Infections in Humans

**DOI:** 10.1371/journal.pntd.0002274

**Published:** 2013-06-13

**Authors:** Andreas Puschnik, Louis Lau, Elizabeth A. Cromwell, Angel Balmaseda, Simona Zompi, Eva Harris

**Affiliations:** 1 Division of Infectious Diseases and Vaccinology, School of Public Health, University of California, Berkeley, Berkeley, California, United States of America; 2 University of North Carolina at Chapel Hill, Chapel Hill, North Carolina, United States of America; 3 Laboratorio Nacional de Virología, Centro Nacional de Diagnóstico y Referencia, Ministry of Health, Managua, Nicaragua; Tropical Medicine Institute Pedro Kourí (IPK), Cuba

## Abstract

Although heterotypic secondary infection with dengue virus (DENV) is associated with severe disease, the majority of secondary infections are mild or asymptomatic. The mechanisms of antibody-mediated protection are poorly understood. In 2010, 108 DENV3-positive cases were enrolled in a pediatric hospital-based study in Managua, Nicaragua, with 61 primary and 47 secondary infections. We analyzed DENV-specific neutralization titers (NT_50_), IgM and IgG avidity, and antibody titer in serum samples collected during acute and convalescent phases and 3, 6, and 18 months post-infection. NT_50_ titers peaked at convalescence and decreased thereafter. IgG avidity to DENV3 significantly increased between convalescent and 3-month time-points in primary DENV infections and between the acute and convalescent phase in secondary DENV infections. While avidity to DENV2, a likely previous infecting serotype, was initially higher than avidity to DENV3 in secondary DENV infections, the opposite relation was observed 3–18 months post-infection. We found significant correlations between IgM avidity and NT_50_ in acute primary cases and between IgG avidity and NT_50_ in secondary DENV infections. In summary, our findings indicate that IgM antibodies likely play a role in early control of DENV infections. IgG serum avidity to DENV, analyzed for the first time in longitudinal samples, switches from targeting mainly cross-reactive serotype(s) to the current infecting serotype over time. Finally, serum avidity correlates with neutralization capacity.

## Author Summary

Dengue is the most common mosquito-borne viral illness in humans, with 3 billion people at risk for infection. Four different dengue virus serotypes (DENV1–4) cause the disease, which can be either inapparent or present with flu-like symptoms (Dengue Fever, aka “breakbone fever”). The disease can be more severe and sometimes fatal, with signs of bleeding and vascular leakage leading to shock (Dengue Hemorrhagic Fever/Dengue Shock Syndrome). No specific treatment or vaccine is available. Understanding how the human immune response develops during a natural infection can be beneficial for future vaccine studies and trials. DENV-specific serum neutralizing capacity may play a role in protection. The neutralization capacity of the serum may depend on the serum avidity against DENV, the amount (or titer) of the anti-DENV antibodies, and the accessibility of the epitopes targeted by these antibodies. Here we show that DENV-specific IgM antibodies likely play a role in neutralization during primary DENV infections and show a correlation between serum avidity and neutralization capacity in secondary DENV infections, with greater avidity to a previously infecting DENV serotype as compared to the current infecting DENV serotype in the early phases of infection, switching over time to the opposite situation.

## Introduction

The four serotypes of the flavivirus dengue virus (DENV1–4) cause the most common mosquito-borne viral disease in humans worldwide, with 50–100 million people infected annually and over 3 billion people at risk [1]. DENV infection can be asymptomatic or cause a spectrum of disease ranging from classical dengue fever (DF) to more severe, life-threatening forms termed dengue hemorrhagic fever (DHF) and dengue shock syndrome (DSS) [2]. Approximately 500,000 dengue patients require hospitalization annually, of whom a large proportion are children [3]. Although several antiviral and vaccine candidates are in various phases of preclinical and clinical evaluation, current treatment remains supportive care [4].

The immune response to primary (1°) DENV infection is characterized by an early IgM response followed by an IgG response with predominantly IgG1 and IgG3 subclasses [5]. Naïve B cells are stimulated and develop into DENV-specific B cells, which either differentiate into memory B cells (MBCs) residing in the secondary lymphoid organs or into plasma cells (PCs) secreting antigen-specific antibodies (Abs). Short-lived PCs are active during acute infection, while long-lived PCs (LLPCs) migrate to the bone marrow and are responsible for long-term humoral immunity [6], [7]. MBCs, which retain antigen-specific Abs at their surface, and LLPCs, which secrete antigen-specific Abs, undergo affinity maturation, and only clones bearing Abs with the highest affinity survive long-term [8]. This process takes several weeks after acute infection and continues despite the absence of circulating antigen. During a secondary (2°) DENV infection, MBCs are rapidly activated [9], [10]. Prior DENV infection provides robust immunity against the homotypic DENV serotype [11], [12]. In contrast, 2° heterotypic infections are associated with a higher incidence of DHF/DSS, possibly attributable in part to antibody-dependent enhancement (ADE), where pre-formed Abs to the 1° infecting serotype bind but do not neutralize the 2° infecting serotype, instead facilitating an increase in viral uptake by Fcγ-receptor bearing cells [13]. In addition to ADE, cross-reactive T cells formed during the 1° DENV infection can be over-activated, potentially contributing to dengue pathogenesis [14], [15]. However, the vast majority of 2° DENV infections are asymptomatic or only result in mild disease [16], suggesting a protective immune response [17].

DENV neutralization requires sufficient levels of neutralizing Abs and the number of Abs bound to a single virion to exceed the threshold of enhancement, which depends on antibody avidity and the accessibility of epitopes on the virus particle [18]. The avidity of anti-flavivirus monoclonal Abs (MAbs) was shown to positively correlate with neutralization activity *in vitro* [19], consistent with lower neutralizing activity observed with MAbs with lower affinity against variant genotypes within a single DENV serotype [20], [21]. However, the relation between neutralizing activity and polyclonal serum avidity is still unclear [9], [22]. Measurement of IgG avidity has been shown to discriminate between 1° and 2° DENV infection in polyclonal serum [23]–[25] but has not been followed longitudinally.

In this study, we analyzed the DENV-specific neutralization capacity and IgM and IgG avidity to DENV of serum samples from our hospital-based study in Managua, Nicaragua. In Nicaragua, one DENV serotype tends to dominate for several years, while other DENV serotypes co-circulate at lower levels. DENV3 was the dominant serotype in 2008–2011 [26]. Prior to this, DENV2 was the predominant serotype between 1999 and 2002 and between 2005 and 2007 [27]–[29], while DENV1 predominated between 2002 and 2005 [30]. DENV4 only circulates at a low level in Nicaragua [31]. We studied both 1° and 2° DENV infections from the acute phase until 18 months (m) post-infection. In 2° infections, we measured neutralizing Ab titers and avidity to DENV3, the currently infecting serotype, and to DENV2, the most prevalent previously circulating serotype and our prototype to assess cross-reactive responses and cross-protection [9], [28]. As both Ab concentration and avidity can play a significant role in virus neutralization [32], we tested possible correlations between IgM and IgG avidity to DENV, total Ab titer and DENV-specific neutralization titer.

## Materials and Methods

### Ethics statement

The protocol for this study was reviewed and approved by the Institutional Review Boards (IRB) of the University of California, Berkeley, and the Nicaraguan Ministry of Health. Parents or legal guardians of all subjects provided written informed consent, and subjects 6 years of age and older provided assent.

### Study population and laboratory tests

Design and execution of the study, inclusion criteria for the study population, and laboratory tests for confirmation of DENV infection in patients have been previously described [9], [33]. Briefly, study enrollment occurred in the Nicaraguan National Pediatric Reference Hospital, Hospital Infantil Manuel de Jesús Rivera (HIMJR), in Managua from August 1, 2010, to January 31, 2011, during the peak dengue season. Inclusion criteria included age between 6 m and 15 years of age. Samples were collected for 3 consecutive days after enrollment (acute), 14–28 days after onset of symptoms (convalescent), and at 3 m, 6 m and 18 m post-illness. DENV infection was confirmed by reverse transcription–polymerase chain reaction (RT-PCR) amplification of viral RNA [34]; isolation of DENV in C6/36 *Aedes albopictus* cells [27]; seroconversion of DENV-specific IgM Abs as measured by IgM capture enzyme-linked immunosorbent assay (ELISA) between acute-phase and convalescent-phase serum samples [35]; and/or a ≥4-fold increase in total Ab titer, as measured by Inhibition ELISA [30], [36], between paired acute- and convalescent-phase serum samples as previously described [26]. 1° and 2° DENV infections were defined by an Ab titer by Inhibition ELISA of <10 or ≥10 in acute-phase samples, respectively, and/or <2,560 or ≥2,560 in convalescent phase samples, respectively [31], [37]. The total Ab titer was measured only during the acute and convalescent phases of the infection, as it decreases substantially thereafter.

### Viruses and cell lines

DENV was propagated in *Aedes albopictus* C6/36 cells as previously described [9]. Cell supernatants were concentrated by centrifugation through Amicon filters (50 kDa, 3,250×g for 20 min at 4°C) or by ultracentrifugation (90,000×g for 2 h at 4°C, Beckman SW28) and resuspended in PBS. DENV2 (strain N172, passage 3) and DENV3 (strain N7236, passage 3) are clinical strains from Nicaraguan patients isolated in the National Virology Laboratory in Managua and passaged minimally. Raji-DC-SIGN-R cells (gift from B. Doranz, Integral Molecular, Philadelphia, PA) were grown in RPMI-1640 medium (Invitrogen) with 5% FBS at 37°C in 5% CO_2_ for use in neutralization assays [38], [39].

### Neutralization assay

Serum samples were heat-inactivated at 56°C for 20 min and then diluted in RPMI-1640 with 10% FBS at pH 8.0 using eight 3-fold dilutions (1∶10–1∶21,870). Neutralization was assessed by flow cytometry using GFP-expressing DENV reporter virus particles (RVPs) as previously described [38], [39]. The percent infection for each serum dilution was calculated in relation to the no-serum control. Data were expressed as percent infection versus log_10_ of the reciprocal serum dilution and fitted to a sigmoidal dose-response curve using GraphPad Prism 5 software (La Jolla, CA) to determine the titer of Ab that achieved a 50% reduction in infection (50% neutralization titer, NT_50_), which is expressed as the reciprocal of the serum dilution.

### Avidity assay

Serum avidity was measured using a modified ELISA protocol with urea washes [22], [23]. DENV3 N7236 was used at a 1∶300 dilution (3.4×10^4^ pfu/ml), which yielded an OD of 1.0 using WHO polyvalent serum. To standardize the amount of DENV2 N172 to the amount of DENV3, we used a pan-DENV MAb, 2H12 (gift from G. Screaton, Imperial College, UK) [40]. Serial dilutions of DENV2 and the 1∶300 dilution of DENV3 were coated and incubated with 1 µg/mL of 2H12 MAb. The 1∶300 dilution of DENV2 (3.6×10^4^ pfu/ml) yielded the same OD as the 1∶300 DENV3 and was used thereafter. To determine serum IgG avidity, plates were coated with whole virus and incubated with heat-inactivated diluted serum samples (1∶100) in triplicate, then washed with urea or PBS for 10 min before adding the secondary biotin-conjugated Ab (donkey anti-human IgG, 1∶1,000), streptavidin-AP conjugate (1∶1,000), and PnPP substrate [9]. Using samples from the same study population, we previously optimized the amount of urea to be used, and 6M urea for 1° DENV infections and 9M urea for 2° DENV infections yielded the best results for analyzing serum avidity to DENV over time [9]. To measure serum IgM avidity, a donkey anti-human IgM, F_c_-fragment-specific MAb (1∶1,000, Jackson ImmunoResearch) was used as secondary Ab. For acute-phase samples, days 4–6 post-onset of symptoms were chosen. Background levels were measured with dengue-negative human sera. For each sample, avidity was calculated as percentage of IgG or IgM bound by dividing the background-adjusted OD after urea washes by the adjusted OD after PBS washes.

Quality control criteria included: background <0.2 OD, WHO polyvalent serum positive control >5X background OD, and WHO positive control of each plate within the mean +/−1 SD of control plate. The control plate was coated with either DENV2 or DENV3 and incubated with WHO polyvalent serum; half the plate was washed with urea (6M or 9M), while the second half was washed with PBS. The mean and SD for the WHO polyvalent positive control was calculated for each urea concentration and each virus as follows:
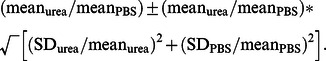



### Statistical analysis

The data were stratified by 1° and 2° infection status for analysis, and gender was evaluated as a possible modifier. Geometric mean total Ab titer, percentage avidity, and NT_50_ were compared using the two-sided Wilcoxon Signed Rank test to detect differences between the following time-points: acute and convalescent; convalescent and 3 m; 3 m and 6 m; and 6 m and 18 m. Geometric mean Ab titer, avidity, and NT_50_ were also compared by infection status at each time-point using the Wilcoxon Signed Rank test. Bivariate correlations between NT_50_ and Ab titer or NT_50_ and avidity were estimated using the Spearman correlation coefficient at each time-point. To test for differences in avidity, NT_50_, and Ab titer by clinical signs of dengue severity (vascular leak, hypotensive shock, compensated shock, cutaneous bleeding, hemoconcentration and mucosal bleeding [37]), two-sided Wilcoxon Signed Rank tests were used to compare the geometric mean NT_50_, total Ab titer and avidity at each time-point, stratifying by immune status. An alpha of p<0.05 was used for statistical significance testing. Calculations were performed in SAS Version 9.2 (The SAS Institute, Cary, NC).

## Results

### Study participants

Between August 1, 2010, and January 31, 2011, 216 patients were enrolled for suspected dengue at the National Pediatric Reference Hospital, HIJMR. Twelve patients were excluded from analysis: one patient dropped out of the study after enrollment and 11 patients had an undetermined dengue diagnostic result. Of the 204 patients who were followed, 108 patients (52.9%) were laboratory-confirmed as DENV3-positive by RT-PCR and/or virus isolation and were included in this analysis (Table S1). Of these, 61 (56.5%) were 1° and 47 (43.5%) were 2° DENV infections (Table 1). Of note, disease severity was relatively low in the 2010–2011 season, with 27 (25.0%) DHF/DSS cases (Table 1) [2]. In the absence of a pre-infection sample, and while we cannot exclude previous DENV1 infections, we hypothesized that most children with 2° DENV infections were previously infected with DENV2, which was the predominant serotype circulating in previous years [28], [29]. Thus, we used DENV2 as a representative previously infecting serotype in our analysis.

**Table 1:** Characteristics of patients enrolled in the hospital-based study during the 2010–2011 dengue season in Managua, Nicaragua.

Indicator	N	%	Mean age ± SD
Participants	108	100	8.4±3.6
Gender			
Female	52	48.2	8.2±3.7
Male	56	51.8	8.7±3.7
Disease classification			
Dengue Fever (DF)	80	74.8	8.1±3.6
Dengue Hemorrhagic Fever (DHF)	25	23.4	9.1±4.0
Dengue Shock Syndrome (DSS)	2	1.9	10.7±0.4
Immune status			
Primary infection	61	56.5	6.9±3.6
Secondary infection	47	43.5	10.4±2.8
Clinical sign (proportion with sign present)*			
Compensated shock	21	19.4	8.5±3.5
Hypotensive shock	6	5.6	8.4±3.8
Vascular leak	71	65.7	7.9±3.8
Cutaneous bleeding	83	77.8	8.2±3.6
Mucosal bleeding	13	12.0	9.6±3.0
Hemoconcentration	3	2.8	9.0±4.7

* Defined as in Narvaez et al [32].

### Longitudinal analysis of DENV-specific serum neutralization

**Figure 1:**
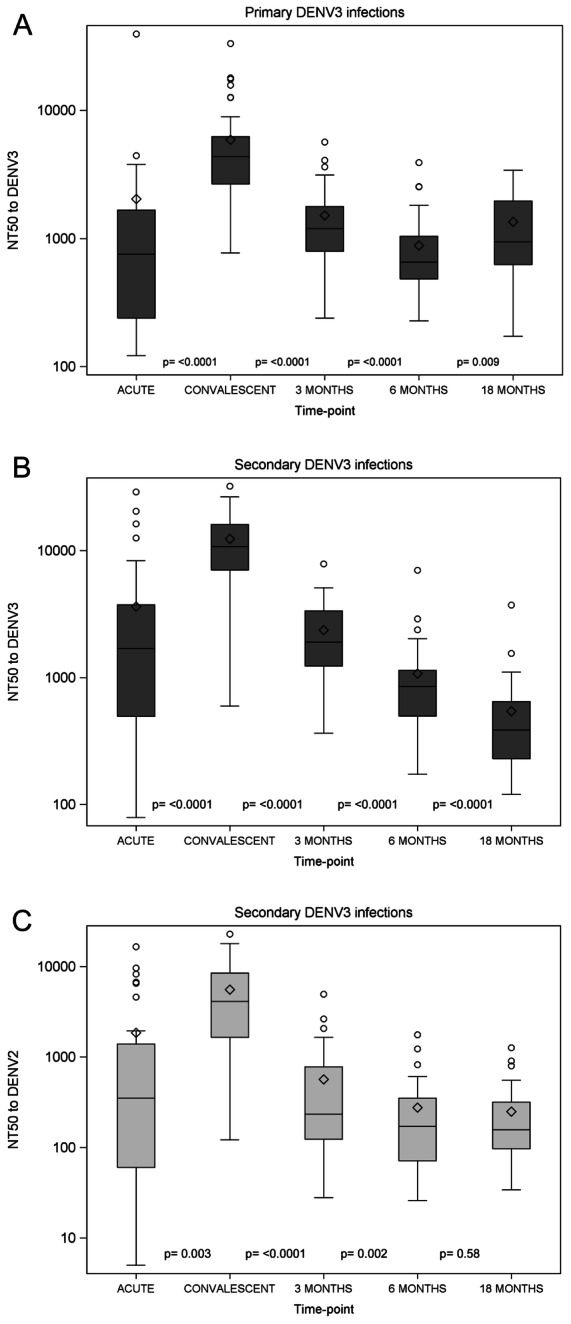
Longitudinal analysis of DENV-specific serum neutralizing Abs. **A.** DENV3-specific NT_50_ of serum day 4–6 (acute), day 14–28 (convalescent), 3 m, 6 m and 18 m post-onset of symptoms in 1° DENV3 infections. Longitudinal serum samples were analyzed via a flow cytometry-based neutralization assay. The mean +/− SD as well as the 25^th^ and 75^th^ percentiles of NT_50_ are displayed. **B.** DENV3-specific NT_50_ of serum day 6 (acute), day 14–28 (convalescent), 3 m, 6 m and 18 m post-onset of symptoms in 2° DENV3 infections. Longitudinal serum samples were analyzed as described in Figure 1A. **C.** DENV2-specific NT_50_ of serum day 6 (acute), day 14–28 (convalescent), 3 m, 6 m and 18 m post-onset of symptoms in 2° DENV3 infections. Longitudinal serum samples were analyzed as described in Figure 1A. Diamonds, mean; middle line, median; upper and lower boundary of the box, intra-quartile range (IQR; 25th to 75th percentile); whiskers, range of values that are outside of the IQR but are close enough not to be considered outliers (within ≤1.5*IQR); empty circles: outliers >1.5*IQR.

We measured the DENV-specific neutralization capacity of patient sera from 1° and 2° DENV3 infections against DENV3, the current infecting serotype, and against DENV2, a representative previously infecting serotype, in 2° infections. Using a flow cytometry-based assay, NT_50_ was determined at acute, convalescent, 3 m, 6 m, and 18 m time-points (Table S2). The NT_50_ against DENV3 peaked at convalescence in both 1° and 2° cases, with significantly higher titers detected in 2° cases (Figure 1A–B). In 1° infections, we observed a significant increase in NT_50_ from the acute to convalescent phase (p<0.001) followed by a significant drop from convalescence to 3 m post-onset of symptoms (p<0.0001), further decreasing at 6 m (p<0.0001) (Figure 1A). For 2° infections, we noted a similar pattern (p<0.0001 for each pair of adjacent time-points) (Figure 1B). In addition, for 2° infections, we measured the DENV2-specific neutralization capacity of the serum, which showed a similar trend (Figure 1C).

### Longitudinal analysis of DENV-specific serum avidity

**Figure 2:**
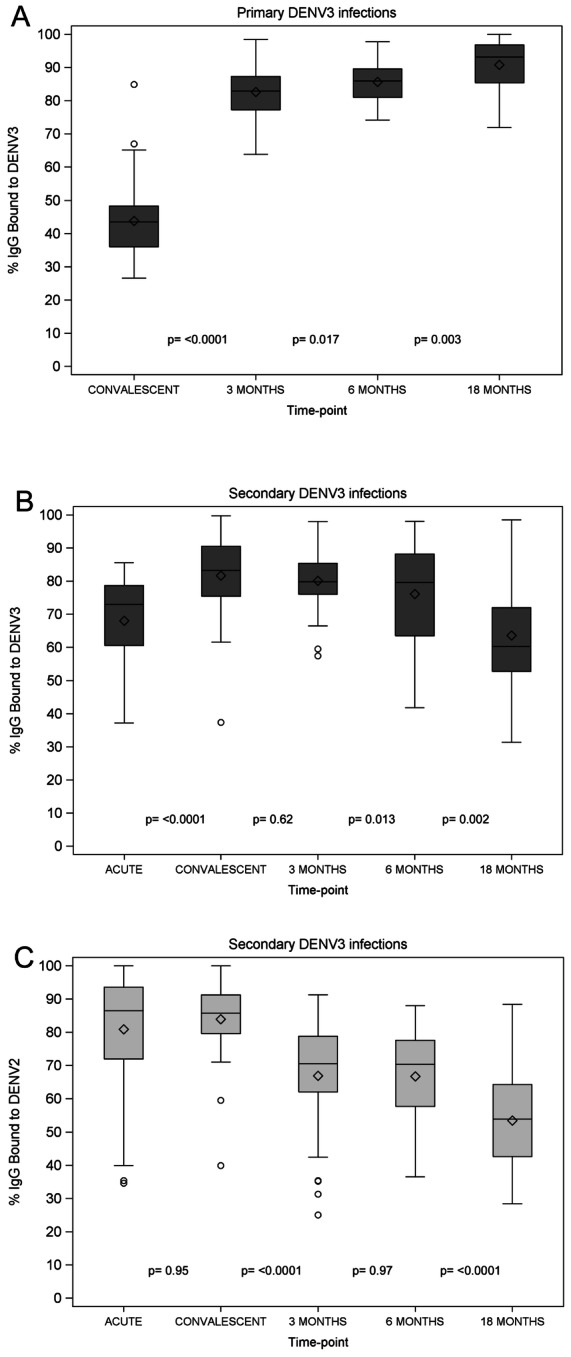
Longitudinal analysis of DENV-specific serum IgG and IgM avidity. **A.** IgG serum avidity to DENV3 day 14–28 (convalescent), 3 m, 6 m and 18 m post-onset of symptoms in 1° DENV3 infections. Longitudinal serum samples were tested for DENV-specific avidity using a modified ELISA with 6M urea washes against DENV3 strain N7236 virions. The mean +/− SD as well as the 25^th^ and 75^th^ percentiles of % IgG bound are displayed. **B.** IgG serum avidity to DENV3 day 6 (acute), day 14–28 (convalescent), 3 m, 6 m and 18 m post-onset of symptoms in 2° DENV3 infections. Longitudinal serum samples were analyzed as in Figure 2A. **C.** DENV3-specific IgG serum avidity day 6 (acute), day 14–28 (convalescent), 3 m, 6 m and 18 m post-onset of symptoms in 2° DENV3 infections. Longitudinal serum samples were analyzed as in Figure 2A. Diamonds, mean; middle line, median; upper and lower boundary of the box, intra-quartile range (IQR; 25th to 75th percentile); whiskers, range of values that are outside of the IQR but are close enough not to be considered outliers (≤1.5*IQR); empty circles, outliers >1.5*IQR.

We measured DENV-specific IgG serum avidity to DENV3 and DENV2 and DENV-specific IgM serum avidity to DENV3 using a modified ELISA with urea washes [22], [23]. Avidity was defined as the percentage (%) of IgG or IgM that remained bound after the urea washes. In 1° DENV3 infections, we observed a large increase in IgG serum avidity to DENV3 between convalescence and 3 m post-onset of symptoms (p<0.0001) and a further rise between 3 and 6 months (p = 0.017) and between 6 and 18 months (p = 0.003) post-onset of symptoms (Figure 2A, Table S2). IgM serum avidity to DENV3 in 1° DENV3 acute samples revealed a mean % IgM bound of 47.9 (SD±12.3). In 2° DENV infections, we observed a significant increase in IgG serum avidity to DENV3 between the acute and convalescent phase (p<0.0001), but no further rise at later time-points (Figure 2B, Table S2).

**Figure 3:**
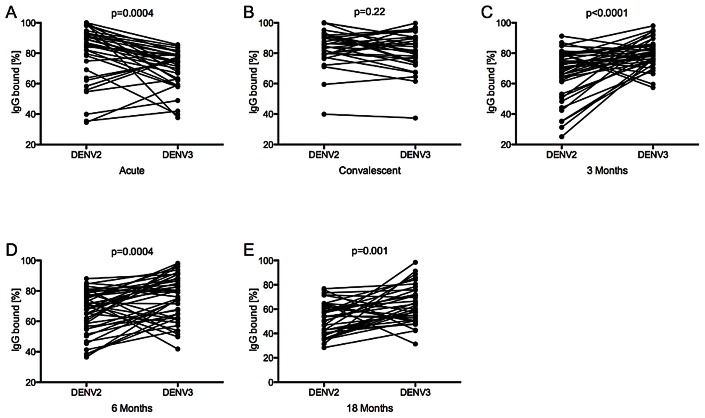
IgG serum avidity to DENV2 versus DENV3 over time. % IgG bound to DENV2 and DENV3 was compared at day 6 (acute) (A), day 14–28 (convalescent) (B), 3 m (C), 6 m (D), and 18 m (E) post-onset of symptoms in 2° DENV3 infections for every patient and is shown as connected dots. The Wilcoxon Rank Sum test was used to compare % IgG bound in serum to DENV2 and DENV3 Nicaraguan viruses.

Measuring the avidity of the same 2° infections to DENV2, a likely previously infecting serotype, we observed significantly higher levels of % IgG bound to DENV2 than DENV3 in the acute phase (p = 0.0004) (Figure 2C, 3A). However, due to the increase in avidity to DENV3 in the convalescent phase, no significant difference in avidity was detectable between the two serotypes (p = 0.22) (Figure 3B). Subsequently, at the 3 m, 6 m, and 18 m time-points, a shift occurred to significantly higher avidity against DENV3, the currently infecting serotype, than against DENV2 (p<0.0001, p = 0.0004, and p = 0.001, respectively) (Figure 3C–E). Of note, the IgG serum avidity remained high for 1° DENV cases, while it declined for 2° cases after the convalescent time-point (Figure 2).

### Correlation between DENV-specific total antibody titer, avidity and neutralization capacity of serum

**Figure 4:**
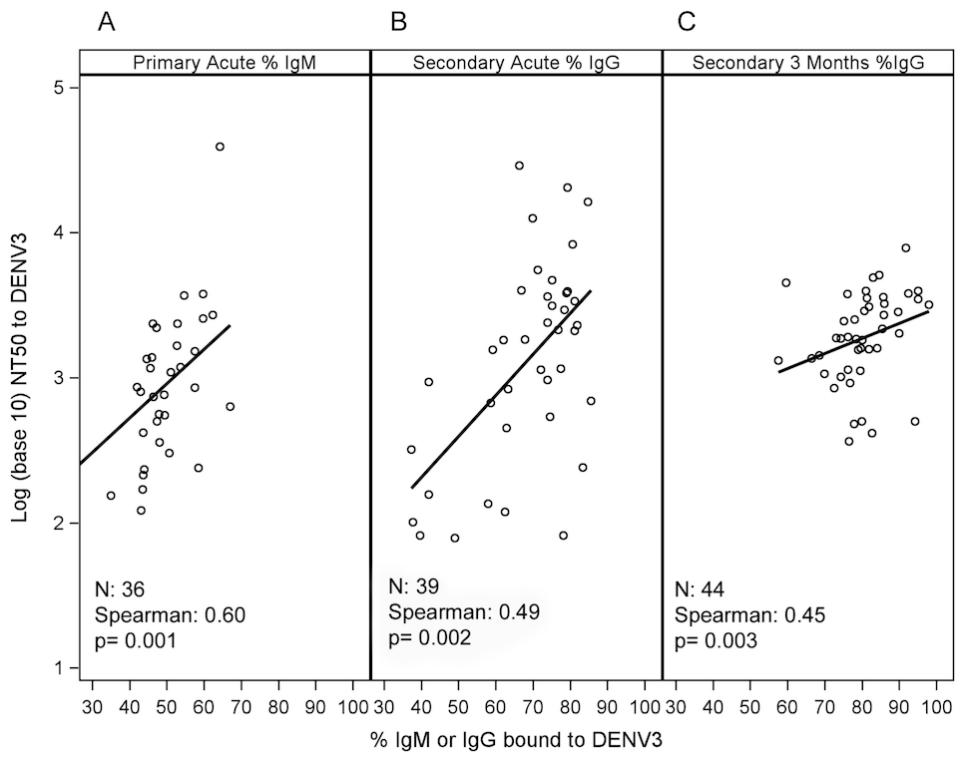
Correlations between DENV-specific serum neutralizing titers and serum avidity over time. Correlation between DENV3-specific NT_50_ and IgM avidity to DENV3 in the acute phase of 1° DENV3 infections (A); correlation between DENV3-specific NT_50_ and IgG avidity to DENV3 in the acute phase of 2° DENV3 infections (B); and correlation between DENV3-specific NT_50_ and IgG avidity to DENV3 3 m post-onset of symptoms in 2° DENV3 infections (C). Spearman's rank correlation coefficient (ρ) and p-value were calculated between the pairs of NT_50_ with % IgM and % IgG bound, respectively.

The ability of serum to neutralize a virus can depend on Ab concentration and/or serum avidity [32], among other parameters. During 1° DENV infections, a positive correlation between IgM serum avidity to DENV3 and DENV3-specific NT_50_ in the acute phase was observed (Figure 4), suggesting contribution of DENV-specific IgM Abs to early virus neutralization. We also observed a positive correlation between IgG serum avidity to DENV3 and NT_50_ to DENV3 in the acute phase of 2° DENV3 cases and at the 3 m time-point (Figure 4, Table 2). Due to the slow kinetics of affinity maturation, these highly avid IgG are most likely secreted by pre-existing MBCs, suggesting the contribution of DENV cross-reactive Ab to virus neutralization. Moreover, a correlation was observed between DENV-specific total Ab titers and NT_50_ against DENV3 in the acute phase of 2° DENV3 infections (Table 2). Interestingly, positive correlations were noted between DENV2-specific NT_50_ and % IgG bound to DENV2 from the acute phase until 18 m post-infection (Figure S1, Table 2). Lastly, a positive correlation was observed between DENV2-specific NT_50_ and IgG serum avidity to DENV3 at acute and convalescent time-points (Figure S2, Table 2).

**Table 2:** Spearman's correlation of NT_50_ and % IgG bound or Ab titer stratified by immune status.

Time-point	N*	Coefficient	p-value
Primary DENV3 % IgG bound vs. DENV3 NT_50_			
Acute	-	-	-
Convalescent	48	0.24	0.095
3 month	48	0.07	0.65
6 month	44	−0.05	0.074
18 month	30	0.11	0.57
Secondary DENV3 % IgG bound vs. DENV3 NT_50_			
Acute	39	**0.49**	**0.0016**
Convalescent	32	−0.08	0.66
3 month	44	**0.45**	**0.003**
6 month	44	0.23	0.13
18 month	37	0.13	0.43
Secondary DENV2 % IgG bound vs. DENV2 NT_50_			
Acute	36	**0.44**	**0.007**
Convalescent	31	**0.44**	**0.013**
3 month	44	**0.42**	**0.004**
6 month	44	**0.57**	**<0.0001**
18 month	38	**0.41**	**0.01**
Secondary DENV2 % IgG bound vs. DENV3 NT_50_			
Acute	35	0.09	0.62
Convalescent	32	0.08	0.66
3 month	44	0.24	0.12
6 month	44	0.08	0.61
18 month	38	0.03	0.87
Secondary DENV3 % IgG bound vs. DENV2 NT_50_			
Acute	40	**0.56**	**0.0002**
Convalescent	31	**0.47**	**0.0073**
3 month	44	0.04	0.8
6 month	44	0.16	0.31
18 month	37	−0.18	0.29
Primary total DENV-specific Ab titer vs. DENV3 NT_50_			
Acute	42	−0.019	0.90
Convalescent	47	0.065	0.66
Secondary total DENV-specific Ab titer vs. DENV3 NT_50_			
Acute	39	**0.61**	**<0.0001**
Convalescent	32	0.32	0.075

* Number of subjects with paired NT_50_, avidity, or Ab titer data for calculation of Spearman's coefficient.

### Association with disease severity and clinical signs of severity

NT_50_, total Ab titer, and % IgG bound were compared between patients with and without the following clinical signs, stratified by immune status: compensated shock, hypotensive shock, vascular leak, cutaneous bleeding, mucosal bleeding, and hemoconcentration (defined as in [33]). No difference was observed in the mean NT_50_, total Ab titer, or % IgG at any time-point examined among 1° or 2° infections. When using the WHO classification (DF vs. DHF/DSS), patients classified as DF did not demonstrate a statistically significant difference in mean NT_50_ or % IgG bound compared to those classified as DHF/DSS (stratified by immune status), except for convalescent % IgG bound to DENV3 in 1° DENV3 infections, with 41.2±8.3 for DF and 53.4±14.2 for DHF/DSS (p = 0.005). A significant difference in total Ab titer was observed between 1° DF and DHF/DSS cases at the convalescent time-point (p = 0.035) with a higher Ab titer observed in more severe cases (248.7±79.9 vs. 109.5±30.9).

## Discussion

A better understanding of protective immune responses to natural DENV infections is critical for the development of safe and effective vaccines and for defining robust correlates of protection for vaccine trials. Protection from DENV infection and/or disease may depend on DENV-specific serum neutralization and thus may be associated with DENV-specific Ab titer and DENV-specific serum avidity. However, several questions remained unanswered, such as the role of IgM Abs and whether serum avidity correlates with serum neutralization. Using our longitudinal sample series from our hospital-based study, we show for the first time that DENV-specific neutralization titers peak in the convalescent phase and then decrease over time in both 1° and 2° DENV3 infections. We also observed increasing DENV3-specific avidity between the convalescent phase and 3 m post-infection in 1° DENV3 cases and between acute and convalescent phases in 2° DENV3 infections. In addition, we detected higher avidity against a heterologous, potentially previously infecting serotype (DENV2) in the acute phase of 2° DENV3 infections, while avidity was higher against the current infecting serotype at later time-points (3–18 m). Finally, we show for the first time a correlation between serum avidity and serum neutralization titers in the context of DENV infection, and specifically demonstrate that serotype-specific neutralizing titers correlate with serum IgM avidity in 1° acute DENV infections and with serum IgG avidity in 2° DENV infections.

In this study, we show that the DENV-specific NT_50_ increases from the acute to the convalescent phase in both 1° and 2° infections, with a higher NT_50_ in 2° cases. Whereas in 1° infections, naïve B cells are stimulated and IgG Abs are detected only in the convalescent phase, cross-reactive memory B cells are reactivated during 2° cases, inducing a rapid increase in DENV-specific IgG Ab in serum [41]. Neutralization is achieved when enough Abs bind to accessible epitopes of DENV, preventing binding to target cells or fusion of the viral and endosomal membranes and subsequent release of viral RNA into the cytoplasm of susceptible cells [42]. At later time-points (3–18 m), a decrease in NT_50_ was observed, as previously shown with DENV and other viruses [41], [43].

In 1° DENV infections, we observed an increase in serum IgG avidity to DENV3 between convalescent phase and 3 m, reflecting affinity maturation of Abs. MBCs and LLPCs develop from naïve B cells and undergo affinity maturation and selection during the first 3 m after infection. As only clones with the highest avidity survive long-term, this leads to a higher mean IgG avidity over time [7], [8]. In contrast, in 2° DENV infections, we observed an increase in serum IgG avidity to DENV3 earlier, between the acute and convalescent phase, which cannot be explained by affinity maturation of newly activated DENV3-specific B cells. Rather, this suggests that cross-reactive Abs secreted by pre-existing MBCs contribute to the early increase in avidity during 2° heterotypic DENV infections.

In previous studies, we found significantly higher serum IgG avidity directed to a possible previously infecting DENV serotype (DENV2) as opposed to the current infecting serotype (DENV3) in the acute phase of 2° DENV3 infections [9]. Here, we confirm these findings and show that subsequently, serum IgG avidity against the current infecting serotype increases over time such that avidity to the current infecting serotype is greater than that to the previous infecting serotype 3–18 m post-infection. Serum IgG avidity to DENV2 decreases at 18 m time-point, possibly as DENV2-specific LLPCs are displaced from the bone marrow and replaced by newly generated DENV3-specific LLPCs. LLPC niches are limited in the bone marrow, and newly formed LLPCs can replace LLPCs formed during earlier infections [44], [45]. The decrease in serum IgG avidity to DENV3 at the 18 m time-point could be explained by a strong extra-follicular response induced by the presence of pre-existing anti-DENV Abs in 2° infections, inhibiting the germinal center response and thus inhibiting a strong long-term immunity to the current infecting serotype, DENV3 [46].

Correlation between serum avidity and serum neutralization has been reported for measles, HIV and cytomegalovirus infections [47]–[49]. We analyzed here whether DENV-specific serum avidity and total Ab titer correlate with DENV-specific neutralization. During 1° DENV infections, neither IgG serum avidity to DENV3 nor DENV-specific total Ab titer correlated with DENV3-specific NT_50_. Rather, the NT_50_ correlated with IgM serum avidity to DENV3 in the acute phase of 1° DENV3 infections, showing that highly avid IgM Abs are also highly neutralizing and suggesting that IgM Abs play an important role in DENV-specific serum neutralization. In contrast, in the acute phase of 2° DENV infections, DENV3-specific NT_50_ correlated with IgG serum avidity to DENV3. Because of the rapid kinetics of appearance of IgG Abs in a 2° DENV infection and the slow affinity maturation process, these highly avid IgG Abs are most likely secreted by MBCs formed during the previous infection rather than by newly-activated naïve B cells. This suggests that cross-reactive Abs contribute to neutralization of acute 2° DENV infections. This is consistent with the isolation of strongly neutralizing cross-reactive MAbs from 2° DENV infections (S. Smith, J. Crowe, R. de Alwis, A. de Silva & E. Harris, unpublished data). IgG serum avidity to DENV2 and DENV2-specific NT_50_ correlated at all time-points, suggesting that affinity maturation after the 1° infection contributes to strengthening the neutralizing activity of serum Abs. We also observed a correlation between IgG serum avidity to DENV3 and DENV2-specific NT_50_, further supporting the relation between cross-reactive Abs and neutralization. Furthermore, a positive correlation between DENV-specific total Ab titer and DENV3-specific NT_50_ in acute 2° DENV infections was found, showing that a greater amount of Abs correlates with neutralization activity.

Since dengue disease severity may be associated with sub-neutralizing (enhancing) concentrations of DENV-specific Abs [50], [51], we analyzed NT_50_ titers and IgG serum avidity among DF vs. DHF/DSS patients and among cases with different clinical signs of severity but did not find many significant differences. However, this could be due to the relatively low disease severity in the 2010–2011 epidemic in Nicaragua and thus the small sample size of DHF/DSS. Finally, pre-infection samples are not available through this hospital-based study; thus, we chose one likely previous infecting serotype (DENV2) to analyze in this group of patients. Analysis of samples collected from documented sequential infections in our long-term prospective dengue cohort study will enable more precise investigation of cross-reactive Abs in 2° DENV infections.

Overall, this study showed that IgM and cross-reactive IgG contribute to neutralization during acute DENV infections. To further support these results, we are conducting additional analyses of the DENV-specific serum neutralization capacity, including: 1) the use of β-mercaptoethanol to chemically deplete IgM Abs and 2) the use of DENV virions to deplete heterologous serotype cross-reactive Abs. While cross-reactive serum avidity dominates the acute 2° DENV response, avidity to the current infecting serotype becomes dominant over time. Significant correlations were observed between neutralizing Ab titers and serum avidity to both the current and a heterotypic serotype. Future studies will address the relation of avidity and NT_50_ to infection outcome (symptomatic vs. inapparent) and to disease severity. The unexpected results from the first proof-of-concept dengue live attenuated vaccine efficacy trial (Phase 2b) that were recently published [52] highlight the critical need to better understand the immune response to natural DENV infections and vaccine candidates and to identify robust correlates of protection. We believe that measurement of DENV-specific serum avidity should be integrated into evaluation of future vaccine trials and applied more broadly to the study of the immune response to DENV after natural infections.

## Supporting Information

Figure S1 **Correlation between DENV2-specific NT_50_ and IgG avidity to DENV2 at all time-points of 2° DENV3 infections.** Spearman's rank correlation coefficient (ρ) and p-value were calculated between the pairs of NT_50_ and % IgG bound.
(DOCX)

Figure S2 **Correlation between DENV2-specific NT_50_ and IgG avidity to DENV3 at acute and convalescent time-points of 2° DENV3 infections.** Spearman's rank correlation coefficient (ρ) and p-value were calculated between the pairs of NT_50_ and % IgG bound.
(DOCX)

Table S1 **Laboratory confirmation of DENV infection.**
(DOCX)

Table S2 **Summary of NT_50_, Ab titer and avidity to DENV3 and DENV2, respectively, by time-point and immune status.**
(DOCX)

## References

[1] Simmons CP, Farrar JJ, Nguyen vV, Wills B (2012) Dengue. N Engl J Med 366: 1423–1432. 22494122 10.1056/NEJMra1110265

[2] WHO (1997) Dengue haemorrhagic fever: diagnosis, treatment, prevention and control. Geneva.

[3] Gibbons RV, Vaughn DW (2002) Dengue: an escalating problem. BMJ 324: 1563–1566. 12089096 10.1136/bmj.324.7353.1563 PMC1123504

[4] Durbin AP, Whitehead SS (2010) Dengue vaccine candidates in development. Curr Top Microbiol Immunol 338: 129–143. 19802583 10.1007/978-3-642-02215-9_10

[5] Murphy BR, Whitehead SS (2011) Immune response to dengue virus and prospects for a vaccine. Annu Rev Immunol 29: 587–619. 21219187 10.1146/annurev-immunol-031210-101315

[6] Amanna IJ, Slifka MK (2010) Mechanisms that determine plasma cell lifespan and the duration of humoral immunity. Immunol Rev 236: 125–138. 20636813 10.1111/j.1600-065X.2010.00912.x PMC7165522

[7] McHeyzer-Williams LJ, McHeyzer-Williams MG (2005) Antigen-specific memory B cell development. Annu Rev Immunol 23: 487–513. 15771579 10.1146/annurev.immunol.23.021704.115732

[8] Smith KG, Light A, Nossal GJ, Tarlinton DM (1997) The extent of affinity maturation differs between the memory and antibody-forming cell compartments in the primary immune response. EMBO J 16: 2996–3006. 9214617 10.1093/emboj/16.11.2996 PMC1169918

[9] Zompi S, Montoya M, Pohl MO, Balmaseda A, Harris E (2012) Dominant cross-reactive B cell response during secondary acute dengue virus infection in humans. PLoS Negl Trop Dis 6: e1568. 22448292 10.1371/journal.pntd.0001568 PMC3308930

[10] Wrammert J, Onlamoon N, Akondy RS, Perng GC, Polsrila K, et al (2012) Rapid and massive virus-specific plasmablast responses during acute dengue virus infection in humans. J Virol 86: 2911–2918. 22238318 10.1128/JVI.06075-11 PMC3302324

[11] Sabin AB (1952) Research on dengue during World War II. Am J Trop Med Hyg 1: 30–50. 14903434 10.4269/ajtmh.1952.1.30

[12] Endy TP, Nisalak A, Chunsuttitwat S, Vaughn DW, Green S, et al (2004) Relationship of preexisting dengue virus (DV) neutralizing antibody levels to viremia and severity of disease in a prospective cohort study of DV infection in Thailand. J Infect Dis 189: 990–1000. 14999601 10.1086/382280

[13] Halstead SB (2003) Neutralization and antibody-dependent enhancement of dengue viruses. Adv Virus Res 60: 421–467. 14689700 10.1016/s0065-3527(03)60011-4

[14] Mangada MM, Rothman AL (2005) Altered cytokine responses of dengue-specific CD4+ T cells to heterologous serotypes. J Immunol 175: 2676–2683. 16081844 10.4049/jimmunol.175.4.2676

[15] Mongkolsapaya J, Dejnirattisai W, Xu XN, Vasanawathana S, Tangthawornchaikul N, et al (2003) Original antigenic sin and apoptosis in the pathogenesis of dengue hemorrhagic fever. Nat Med 9: 921–927. 12808447 10.1038/nm887

[16] Kyle JL, Harris E (2008) Global spread and persistence of dengue. Annu Rev Microbiol 62: 71–92. 18429680 10.1146/annurev.micro.62.081307.163005

[17] Kochel TJ, Watts DM, Halstead SB, Hayes CG, Espinoza A, et al (2002) Effect of dengue-1 antibodies on American dengue-2 viral infection and dengue haemorrhagic fever. Lancet 360: 310–312. 12147378 10.1016/S0140-6736(02)09522-3

[18] Dowd KA, Pierson TC (2011) Antibody-mediated neutralization of flaviviruses: a reductionist view. Virology 411: 306–315. 21255816 10.1016/j.virol.2010.12.020 PMC3100196

[19] Pierson TC, Xu Q, Nelson S, Oliphant T, Nybakken GE, et al (2007) The stoichiometry of antibody-mediated neutralization and enhancement of West Nile virus infection. Cell Host Microbe 1: 135–145. 18005691 10.1016/j.chom.2007.03.002 PMC2656919

[20] Brien JD, Austin SK, Sukupolvi-Petty S, O'Brien KM, Johnson S, et al (2010) Genotype-specific neutralization and protection by antibodies against dengue virus type 3. J Virol 84: 10630–10643. 20702644 10.1128/JVI.01190-10 PMC2950583

[21] Wahala WM, Donaldson EF, de Alwis R, Accavitti-Loper MA, Baric RS, et al (2010) Natural strain variation and antibody neutralization of dengue serotype 3 viruses. PLoS Pathog 6: e1000821. 20333252 10.1371/journal.ppat.1000821 PMC2841629

[22] Zompi S, Santich BH, Beatty PR, Harris E (2012) Protection from secondary dengue virus infection in a mouse model reveals the role of serotype cross-reactive B and T cells. J Immunol 188: 404–416. 22131327 10.4049/jimmunol.1102124 PMC3244532

[23] de Souza VA, Fernandes S, Araujo ES, Tateno AF, Oliveira OM, et al (2004) Use of an immunoglobulin G avidity test to discriminate between primary and secondary dengue virus infections. J Clin Microbiol 42: 1782–1784. 15071049 10.1128/JCM.42.4.1782-1784.2004 PMC387572

[24] Matheus S, Deparis X, Labeau B, Lelarge J, Morvan J, et al (2005) Use of four dengue virus antigens for determination of dengue immune status by enzyme-linked immunosorbent assay of immunoglobulin G avidity. J Clin Microbiol 43: 5784–5786. 16272520 10.1128/JCM.43.11.5784-5786.2005 PMC1287789

[25] Matheus S, Deparis X, Labeau B, Lelarge J, Morvan J, et al (2005) Discrimination between primary and secondary dengue virus infection by an immunoglobulin G avidity test using a single acute-phase serum sample. J Clin Microbiol 43: 2793–2797. 15956399 10.1128/JCM.43.6.2793-2797.2005 PMC1151893

[26] Gutierrez G, Standish K, Narvaez F, Perez MA, Saborio S, et al (2011) Unusual dengue virus 3 epidemic in Nicaragua, 2009. PLoS Negl Trop Dis 5: e1394. 22087347 10.1371/journal.pntd.0001394 PMC3210753

[27] Balmaseda A, Sandoval E, Perez L, Gutierrez CM, Harris E (1999) Application of molecular typing techniques in the 1998 dengue epidemic in Nicaragua. Am J Trop Med Hyg 61: 893–897. 10674666 10.4269/ajtmh.1999.61.893

[28] OhAinle M, Balmaseda A, Macalalad AR, Tellez Y, Zody MC, et al (2011) Dynamics of dengue disease severity determined by the interplay between viral genetics and serotype-specific immunity. Sci Transl Med 3: 114ra128. 10.1126/scitranslmed.3003084 PMC4517192 22190239

[29] Hammond SN, Balmaseda A, Perez L, Tellez Y, Saborio SI, et al (2005) Differences in dengue severity in infants, children, and adults in a 3-year hospital-based study in Nicaragua. Am J Trop Med Hyg 73: 1063–1070. 16354813

[30] Balmaseda A, Hammond SN, Tellez Y, Imhoff L, Rodriguez Y, et al (2006) High seroprevalence of antibodies against dengue virus in a prospective study of schoolchildren in Managua, Nicaragua. Trop Med Int Health 11: 935–942. 16772016 10.1111/j.1365-3156.2006.01641.x

[31] Balmaseda A, Hammond SN, Perez L, Tellez Y, Saborio SI, et al (2006) Serotype-specific differences in clinical manifestations of dengue. Am J Trop Med Hyg 74: 449–456. 16525106

[32] Bachmann MF, Kalinke U, Althage A, Freer G, Burkhart C, et al (1997) The role of antibody concentration and avidity in antiviral protection. Science 276: 2024–2027. 9197261 10.1126/science.276.5321.2024

[33] Narvaez F, Gutierrez G, Perez MA, Elizondo D, Nunez A, et al (2011) Evaluation of the traditional and revised WHO classifications of Dengue disease severity. PLoS Negl Trop Dis 5: e1397. 22087348 10.1371/journal.pntd.0001397 PMC3210746

[34] Lanciotti RS, Calisher CH, Gubler DJ, Chang GJ, Vorndam AV (1992) Rapid detection and typing of dengue viruses from clinical samples by using reverse transcriptase-polymerase chain reaction. J Clin Microbiol 30: 545–551. 1372617 10.1128/jcm.30.3.545-551.1992 PMC265106

[35] Balmaseda A, Guzman MG, Hammond S, Robleto G, Flores C, et al (2003) Diagnosis of dengue virus infection by detection of specific immunoglobulin M (IgM) and IgA antibodies in serum and saliva. Clin Diagn Lab Immunol 10: 317–322. 12626461 10.1128/CDLI.10.2.317-322.2003 PMC150529

[36] Fernandez RJ, Vazquez S (1990) Serological diagnosis of dengue by an ELISA inhibition method (EIM). Mem Inst Oswaldo Cruz 85: 347–351. 2134709 10.1590/s0074-02761990000300012

[37] Harris E, Videa E, Perez L, Sandoval E, Tellez Y, et al (2000) Clinical, epidemiologic, and virologic features of dengue in the 1998 epidemic in Nicaragua. Am J Trop Med Hyg 63: 5–11. 11357995 10.4269/ajtmh.2000.63.5

[38] Ansarah-Sobrinho C, Nelson S, Jost CA, Whitehead SS, Pierson TC (2008) Temperature-dependent production of pseudoinfectious dengue reporter virus particles by complementation. Virology 381: 67–74. 18801552 10.1016/j.virol.2008.08.021 PMC3428711

[39] Mattia K, Puffer BA, Williams KL, Gonzalez R, Murray M, et al (2011) Dengue reporter virus particles for measuring neutralizing antibodies against each of the four dengue serotypes. PLoS One 6: e27252. 22096543 10.1371/journal.pone.0027252 PMC3212561

[40] Midgley CM, Bajwa-Joseph M, Vasanawathana S, Limpitikul W, Wills B, et al (2011) An in-depth analysis of original antigenic sin in dengue virus infection. J Virol 85: 410–421. 20980526 10.1128/JVI.01826-10 PMC3014204

[41] Vaughn DW, Green S, Kalayanarooj S, Innis BL, Nimmannitya S, et al (1997) Dengue in the early febrile phase: viremia and antibody responses. J Infect Dis 176: 322–330. 9237696 10.1086/514048

[42] Pierson TC, Diamond MS (2008) Molecular mechanisms of antibody-mediated neutralisation of flavivirus infection. Expert Rev Mol Med 10: e12. 18471342 10.1017/S1462399408000665 PMC2671962

[43] Liu L, Xie J, Sun J, Han Y, Zhang C, et al (2011) Longitudinal profiles of immunoglobulin G antibodies against severe acute respiratory syndrome coronavirus components and neutralizing activities in recovered patients. Scand J Infect Dis 43: 515–521. 21366405 10.3109/00365548.2011.560184

[44] Odendahl M, Mei H, Hoyer BF, Jacobi AM, Hansen A, et al (2005) Generation of migratory antigen-specific plasma blasts and mobilization of resident plasma cells in a secondary immune response. Blood 105: 1614–1621. 15507523 10.1182/blood-2004-07-2507

[45] Radbruch A, Muehlinghaus G, Luger EO, Inamine A, Smith KG, et al (2006) Competence and competition: the challenge of becoming a long-lived plasma cell. Nat Rev Immunol 6: 741–750. 16977339 10.1038/nri1886

[46] Paus D, Phan TG, Chan TD, Gardam S, Basten A, et al (2006) Antigen recognition strength regulates the choice between extrafollicular plasma cell and germinal center B cell differentiation. J Exp Med 203: 1081–1091. 16606676 10.1084/jem.20060087 PMC2118299

[47] Nair N, Moss WJ, Scott S, Mugala N, Ndhlovu ZM, et al (2009) HIV-1 infection in Zambian children impairs the development and avidity maturation of measles virus-specific immunoglobulin G after vaccination and infection. J Infect Dis 200: 1031–1038. 19702505 10.1086/605648 PMC2938771

[48] Bower JF, Li Y, Wyatt R, Ross TM (2006) HIV-1 Envgp140 trimers elicit neutralizing antibodies without efficient induction of conformational antibodies. Vaccine 24: 5442–5451. 16621193 10.1016/j.vaccine.2006.03.063

[49] Nozawa N, Fang-Hoover J, Tabata T, Maidji E, Pereira L (2009) Cytomegalovirus-specific, high-avidity IgG with neutralizing activity in maternal circulation enriched in the fetal bloodstream. J Clin Virol 46 Suppl 4: S58–63. 19854676 10.1016/j.jcv.2009.10.004 PMC2794836

[50] Beltramello M, Williams KL, Simmons CP, Macagno A, Simonelli L, et al (2010) The human immune response to Dengue virus is dominated by highly cross-reactive antibodies endowed with neutralizing and enhancing activity. Cell Host Microbe 8: 271–283. 20833378 10.1016/j.chom.2010.08.007 PMC3884547

[51] Shrestha B, Brien JD, Sukupolvi-Petty S, Austin SK, Edeling MA, et al (2010) The development of therapeutic antibodies that neutralize homologous and heterologous genotypes of dengue virus type 1. PLoS Pathog 6: e1000823. 20369024 10.1371/journal.ppat.1000823 PMC2848552

[52] Sabchareon A, Wallace D, Sirivichayakul C, Limkittikul K, Chanthavanich P, et al (2012) Protective efficacy of the recombinant, live-attenuated, CYD tetravalent dengue vaccine in Thai schoolchildren: a randomised, controlled phase 2b trial. Lancet 380: 1559–1567. 22975340 10.1016/S0140-6736(12)61428-7

