# Supplementary figures and images for: Correlation between Dengue-Specific Neutralizing Antibodies and Serum Avidity in Primary and Secondary Dengue Virus 3 Natural Infections in Humans
Source: PLoS Negl Trop Dis. 2013 Jun 13;7(6):e2274. doi: 10.1371/journal.pntd.0002274 (PMC3681624; doi:10.1371/journal.pntd.0002274)

**
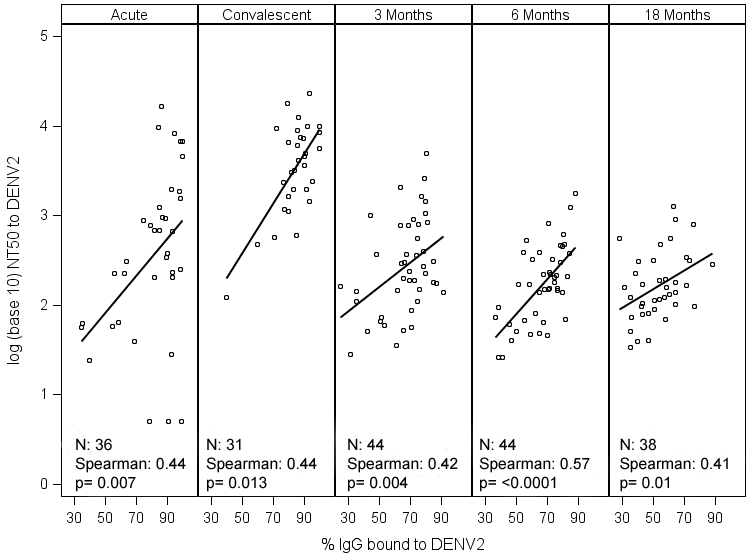
**

Supplement: Figure S1 — Correlation between DENV2-specific NT50 and IgG avidity to DENV2 at all time-points of 2° DENV3 infections. Spearman's rank correlation coefficient (ρ) and p-value were calculated between the pairs of NT50 and % IgG bound. (DOCX) [file pntd.0002274.s001.docx]

**
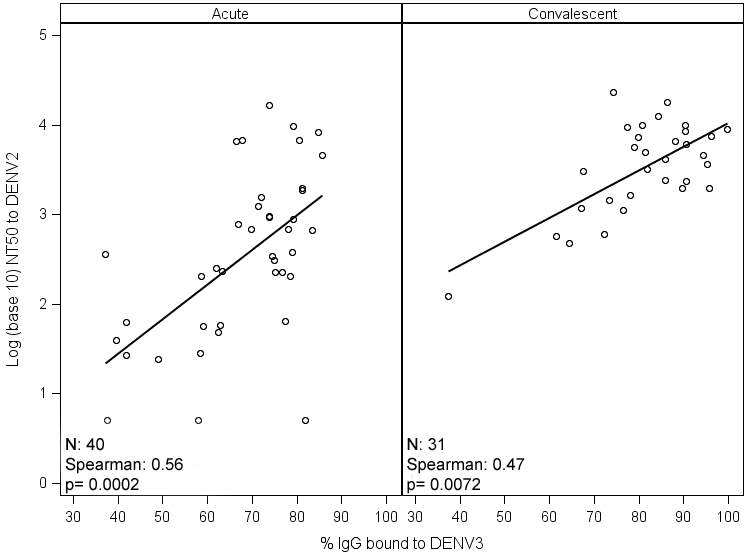
**

Supplement: Figure S2 — Correlation between DENV2-specific NT50 and IgG avidity to DENV3 at acute and convalescent time-points of 2° DENV3 infections. Spearman's rank correlation coefficient (ρ) and p-value were calculated between the pairs of NT50 and % IgG bound. (DOCX) [file pntd.0002274.s002.docx]
