# Supplementary material for: Correlation between Dengue-Specific Neutralizing Antibodies and Serum Avidity in Primary and Secondary Dengue Virus 3 Natural Infections in Humans
Source: PLoS Negl Trop Dis. 2013 Jun 13;7(6):e2274. doi: 10.1371/journal.pntd.0002274 (PMC3681624; doi:10.1371/journal.pntd.0002274)
Supplement: Table S1 — Laboratory confirmation of DENV infection. (DOCX) [file pntd.0002274.s003.docx]

| **Patient** | **Infection** | **DENV** | **DENV IgM acute phase** | **DENV IgM conv. phase** | **Inhibition ELISA acute phase** | **Inhibition ELISA conv. phase** | **DENV serotype by PCR** | **DENV serotype by virus isolation** |
| --- | --- | --- | --- | --- | --- | --- | --- | --- |
| 1017 | primary | 3 | negative | positive | <10 | 322 | 3 | 3 |
| 1019 | primary | 3 | negative | positive | <10 | 87 | 3 | 3 |
| 1021 | primary | 3 | negative | positive | <10 | 29 | 3 | 3 |
| 1023 | primary | 3 | negative | positive | <10 | 104 | 3 | -* |
| 1024 | primary | 3 | negative | positive | <10 | 39 | 3 | 3 |
| 1025 | primary | 3 | positive | NC** | <10 | NC** | 3 | -* |
| 1026 | primary | 3 | negative | negative | <10 | 25 | 3 | 3 |
| 1040 | primary | 3 | negative | positive | <10 | 222 | 3 | -* |
| 1053 | primary | 3 | negative | NC** | <10 | NC** | 3 | 3 |
| 1067 | primary | 3 | negative | negative | <10 | 101 | 3 | 3 |
| 1068 | primary | 3 | negative | positive | <10 | 104 | 3 | 3 |
| 1071 | primary | 3 | negative | positive | <10 | 35 | 3 | -* |
| 1073 | primary | 3 | negative | positive | <10 | 460 | 3 | 3 |
| 1074 | primary | 3 | negative | positive | <10 | 75 | 3 | 3 |
| 1076 | primary | 3 | negative | positive | <10 | 101 | 3 | 3 |
| 1091 | primary | 3 | positive | positive | <10 | 27 | 3 | -* |
| 1092 | primary | 3 | negative | positive | <10 | 91 | 3 | 3 |
| 1093 | primary | 3 | positive | negative | 18 | 2171 | 3 | -* |
| 1103 | primary | 3 | negative | positive | <10 | 274 | 3 | 3 |
| 1104 | primary | 3 | negative | positive | <10 | 14 | 3 | 3 |
| 1105 | primary | 3 | negative | positive | <10 | 31 | 3 | -* |
| 1106 | primary | 3 | negative | positive | <10 | 22 | 3 | 3 |
| 1108 | primary | 3 | positive | positive | <10 | 155 | 3 | -* |
| 1109 | primary | 3 | positive | NC** | <10 | NC** | 3 | -* |
| 1115 | primary | 3 | positive | positive | <10 | 27 | 3 | -* |
| 1116 | primary | 3 | negative | NC** | <10 | NC** | 3 | 3 |
| 1117 | primary | 3 | positive | positive | <10 | 62 | 3 | -* |
| 1119 | primary | 3 | negative | positive | <10 | 90 | 3 | 3 |
| 1122 | primary | 3 | negative | positive | <10 | 22 | 3 | 3 |
| 1126 | primary | 3 | negative | positive | <10 | 43 | 3 | 3 |
| 1128 | primary | 3 | negative | positive | <10 | <10 | 3 | 3 |
| 1129 | primary | 3 | negative | positive | <10 | 12 | -* | 3 |
| 1135 | primary | 3 | positive | positive | <10 | 186 | 3 | -* |
| 1136 | primary | 3 | negative | positive | <10 | 132 | 3 | 3 |
| 1143 | primary | 3 | negative | positive | 18 | 662 | 3 | 3 |
| 1149 | primary | 3 | negative | positive | <10 | 1084 | 3 | 3 |
| 1150 | primary | 3 | negative | positive | <10 | 15 | 3 | -* |
| 1151 | primary | 3 | negative | positive | <10 | 116 | 3 | 3 |
| 1165 | primary | 3 | negative | positive | <10 | 14 | 3 | 3 |
| 1167 | primary | 3 | negative | positive | <10 | 39 | 3 | -* |
| 1171 | primary | 3 | negative | positive | <10 | 22 | 3 | 3 |
| 1172 | primary | 3 | negative | positive | <10 | 118 | 3 | 3 |
| 1174 | primary | 3 | negative | positive | <10 | 164 | 3 | 3 |
| 1175 | primary | 3 | negative | positive | <10 | 125 | 3 | 3 |
| 1182 | primary | 3 | negative | negative | <10 | 131 | 3 | -* |
| 1183 | primary | 3 | positive | positive | <10 | 586 | 3 | 3 |
| 1185 | primary | 3 | negative | positive | <10 | 109 | 3 | 3 |
| 1190 | primary | 3 | positive | positive | <10 | 55 | 3 | 3 |
| 1192 | primary | 3 | negative | positive | <10 | 39 | 3 | 3 |
| 1193 | primary | 3 | negative | positive | <10 | 591 | 3 | -* |
| 1195 | primary | 3 | negative | positive | <10 | 11 | 3 | 3 |
| 1196 | primary | 3 | negative | positive | <10 | 25 | 3 | 3 |
| 1202 | primary | 3 | negative | positive | <10 | 192 | 3 | 3 |
| 1213 | primary | 3 | negative | positive | <10 | 28 | 3 | 3 |
| 1214 | primary | 3 | positive | positive | <10 | 27 | 3 | 3 |
| 1220 | primary | 3 | negative | positive | <10 | 20 | 3 | -* |
| 1224 | primary | 3 | negative | positive | <10 | 42674 | 3 | 3 |
| 1225 | primary | 3 | positive | positive | <10 | 63 | 3 | 3 |
| 1227 | primary | 3 | positive | positive | <10 | 93 | -* | 3 |
| 1228 | primary | 3 | negative | positive | <10 | 380 | 3 | 3 |
| 1231 | primary | 3 | negative | positive | <10 | 353 | 3 | 3 |
| 1028 | secondary | 3 | negative | negative | 372 | 52242 | 3 | 3 |
| 1031 | secondary | 3 | positive | positive | 1772 | 39082 | 3 | 3 |
| 1034 | secondary | 3 | negative | positive | 451 | 58258 | 3 | 3 |
| 1042 | secondary | 3 | negative | negative | 31 | 41264 | 3 | 3 |
| 1044 | secondary | 3 | negative | positive | 1884 | 31765 | 3 | 3 |
| 1051 | secondary | 3 | positive | positive | 37021 | 51563 | 3 | -* |
| 1054 | secondary | 3 | negative | positive | 809 | 45145 | 3 | 3 |
| 1060 | secondary | 3 | negative | positive | 51 | 36905 | 3 | 3 |
| 1082 | secondary | 3 | negative | negative | 150 | 43268 | 3 | 3 |
| 1083 | secondary | 3 | negative | positive | 150 | 43268 | 3 | 3 |
| 1085 | secondary | 3 | negative | negative | 364 | 3467 | 3 | -* |
| 1086 | secondary | 3 | negative | positive | 3741 | >100000 | 3 | -* |
| 1090 | secondary | 3 | positive | positive | 3494 | 25508 | 3 | -* |
| 1095 | secondary | 3 | negative | positive | 130 | >100000 | 3 | 3 |
| 1101 | secondary | 3 | negative | positive | 1890 | 16492 | 3 | -* |
| 1110 | secondary | 3 | negative | positive | 30 | 28118 | 3 | 3 |
| 1114 | secondary | 3 | positive | positive | 7459 | 64390 | 3 | -* |
| 1123 | secondary | 3 | negative | positive | 1667 | 59862 | 3 | -* |
| 1124 | secondary | 3 | negative | positive | 37 | 22478 | 3 | 3 |
| 1127 | secondary | 3 | positive | positive | 9014 | 15012 | 3 | -* |
| 1130 | secondary | 3 | negative | positive | 293 | >100000 | 3 | 3 |
| 1132 | secondary | 3 | negative | positive | 302 | 82656 | 3 | -* |
| 1133 | secondary | 3 | negative | positive | 61 | 7469 | 3 | -* |
| 1134 | secondary | 3 | negative | positive | 3782 | 45789 | 3 | -* |
| 1140 | secondary | 3 | negative | positive | 154 | >100000 | 3 | 3 |
| 1142 | secondary | 3 | negative | positive | 265 | >100000 | 3 | 3 |
| 1147 | secondary | 3 | negative | positive | 61 | 37269 | 3 | 3 |
| 1148 | secondary | 3 | negative | positive | 305 | 22890 | 3 | 3 |
| 1153 | secondary | 3 | negative | positive | 673 | 32539 | 3 | 3 |
| 1155 | secondary | 3 | negative | negative | 407 | 12923 | 3 | 3 |
| 1159 | secondary | 3 | negative | negative | 932 | 1552 | 3 | 3 |
| 1162 | secondary | 3 | negative | negative | 184 | 56706 | 3 | -* |
| 1168 | secondary | 3 | negative | negative | 2328 | 16695 | 3 | 3 |
| 1169 | secondary | 3 | negative | positive | 2844 | >100000 | 3 | 3 |
| 1170 | secondary | 3 | negative | positive | <10 | >100000 | 3 | 3 |
| 1177 | secondary | 3 | positive | positive | 1499 | 77906 | 3 | 3 |
| 1178 | secondary | 3 | negative | positive | 33 | 58126 | 3 | 3 |
| 1181 | secondary | 3 | negative | negative | 410 | 39029 | 3 | 3 |
| 1184 | secondary | 3 | negative | positive | 137 | 27856 | 3 | 3 |
| 1187 | secondary | 3 | negative | positive | 150 | 13361 | 3 | 3 |
| 1191 | secondary | 3 | negative | positive | 16 | 25880 | 3 | 3 |
| 1194 | secondary | 3 | positive | positive | 43 | 42987 | 3 | 3 |
| 1209 | secondary | 3 | negative | positive | 38 | 62669 | 3 | 3 |
| 1212 | secondary | 3 | negative | positive | 287 | 37915 | 3 | 3 |
| 1219 | secondary | 3 | negative | positive | 1872 | 30197 | 3 | 3 |
| 1221 | secondary | 3 | negative | positive | 451 | 14376 | 3 | 3 |
| 1230 | secondary | 3 | negative | positive | 5573 | >100000 | -* | 3 |

*, negative test result

**, no convalescent sample available
