# Supplementary material for: Correlation between Dengue-Specific Neutralizing Antibodies and Serum Avidity in Primary and Secondary Dengue Virus 3 Natural Infections in Humans
Source: PLoS Negl Trop Dis. 2013 Jun 13;7(6):e2274. doi: 10.1371/journal.pntd.0002274 (PMC3681624; doi:10.1371/journal.pntd.0002274)
Supplement: Table S2 — Summary of NT50, Ab titer and avidity to DENV3 and DENV2, respectively, by time-point and immune status. (DOCX) [file pntd.0002274.s004.docx]

| **Summary of NT_50_, Ab titer and avidity to DENV3 results by time-point and immune status** | | | | | | | | | | | | | | | | | | | | | | | | | | | | |
| --- | --- | --- | --- | --- | --- | --- | --- | --- | --- | --- | --- | --- | --- | --- | --- | --- | --- | --- | --- | --- | --- | --- | --- | --- | --- | --- | --- | --- |
| Primary DENV infections | | | | | | | | | | | | | | | | | | | | | | | | | | | | |
| Indicator |  |  | Acute | |  |  | Convalescent | | | | |  | |  | | 3 Months | |  |  | 6 Months | |  |  | 18 Months | | | |  |
|  |  | N | Mean | SD |  | N | Mean | | SD | | |  | | N | | Mean | SD |  | N | Mean | SD |  | N | Mean | | SD | |  |
| NT_50_ |  | 42 | 955.1 | 4.2 |  | 48 | 6166.4 | | 2.3 | | |  | | 48 | | 1510.0 | 2.1 |  | 44 | 769.3 | 1.9 |  | 31 | 613.9 | | 2.4 | |  |
| % IgG bound |  | - | - | - |  | 48 | 43.8 | | 10.9 | | |  | | 48 | | 82.6 | 7.2 |  | 45 | 85.7 | 6.0 |  | 33 | 90.8 | | 7.9 | |  |
| Antibody titer |  | 61 | 5.4 | 2.3 |  | 47 | 136.1 | | 205.6 | | |  | | - | | - | - |  | - | - | - |  | - | - | | - | |  |
| Secondary DENV infections | | | | | | | | | | | | | | | | | | | | | | | | | | | | |
| NT_50_ |  | 40 | 1281.1 | 4.9 |  | 32 | 10210.5 | | 2.1 | | |  | | 44 | | 1885.8 | 20.7 |  | 44 | 825.1 | 2.0 |  | 39 | 405.0 | | 2.0 | |  |
| % IgG bound |  | 40 | 68.0 | 13.8 |  | 32 | 81.67 | | 12.9 | | |  | | 45 | | 80.1 | 8.8 |  | 44 | 76.1 | 14.8 |  | 37 | 63.6 | | 15.1 | |  |
| Antibody titer |  | 46 | 1230.6 | 1970.3 |  | 36 | 53369.7 | | 30147.7 | | |  | | - | | - | - |  | - | - | - |  | - | - | | - | |  |
|  |  | |  |  |  |  |  |  |  | |  | | | | |  |  |  |  |  |  |  |  |  |  | |  |  |
| **Summary of NT_50_, Ab titer and avidity to DENV2 results by time-point among secondary DENV infections** | | | | | | | | | | | | | | | | | | | | | | | | | | | | |
| Indicator |  |  | Acute | |  |  | Convalescent | | |  | | |  | | 3 Months | | |  |  | 6 Months | |  |  | 18 Months | | | |  |
|  |  | N | Mean | SD |  | N | Mean | | SD | | |  | | N | | Mean | SD |  | N | Mean | SD |  | N | Mean | | SD | |  |
| NT_50_ |  | 40 | 342.5 | 8.6 |  | 31 | 3353.3 | | 3.2 | | |  | | 44 | | 268.7 | 3.4 |  | 44 | 170.2 | 2.7 |  | 39 | 167.9 | | 40 | |  |
| % IgG bound |  | 36 | 80.9 | 18.7 |  | 32 | 83.9 | | 12.0 | | |  | | 45 | | 66.9 | 15.8 |  | 44 | 66.7 | 13.9 |  | 38 | 53.4 | | 14.7 | |  |
| Antibody titer |  | 46 | 1230.6 | 1970.3 |  | 36 | 53369.7 | | 30147.7 | | |  | | - | | - | - |  | - | - | - |  | - | - | | - | |  |
